# Decitabine attenuates ischemic stroke by reducing astrocytes proliferation in rats

**DOI:** 10.1371/journal.pone.0272482

**Published:** 2022-08-02

**Authors:** Qi Zhang, Dan Li, Haihua Zhao, Xu Zhang

**Affiliations:** Department of Human Anatomy, College of Basic Medical Sciences, China Medical University, Shenyang, China; Barrow Neurological Institute, UNITED STATES

## Abstract

DNA methylation regulates epigenetic gene expression in ischemic stroke. Decitabine attenuates ischemic stroke by inhibiting DNA methylation. However, the underlying mechanism of this effect is not known. A model of ischemic stroke in Sprague-Dawley rats was induced through middle cerebral artery occlusion followed by reperfusion step. The rats were randomly treated with decitabine or vehicle by a one-time intraperitoneal injection. Sham rats received similar treatments. Four days after treatment, the rats were perfused with saline or 4% paraformaldehyde after which the brain was excised. DNA methylation level and brain infarct volume were determined by dot blot and histochemistry, respectively. The cellular co-localization and quantitative analysis of DNA methylation were assessed by immunohistochemistry and expression levels of cdkn1b (p27) mRNA and protein were measured by qRT-PCR and immunohistochemistry, respectively. The proliferation of astrocytes and number of neurons were determined by immunohistochemistry. Rats treated with decitabine showed hypomethylation and reduced infarct volume in the cortex. DNA methylation was decreased in astrocytes. Decitabine upregulated p27 mRNA and protein expression levels and attenuated the proliferation of astrocytes in vivo and vitro. Decitabine promotes p27 gene expression possibly by inhibiting its DNA methylation, thereby decreases the proliferation of astrocytes, neuronal death and infarct volume after ischemic stroke.

## Introduction

DNA methylation is an epigenetic mechanism through which DNA methyltransferases (Dnmts) regulate gene expression by transfer of methyl group to cytosine residues in gene-regulatory regions [1, 2]. This type of modification can therefore affect the cell differentiation and development in almost all tissue. It is a heritable modification without changing the specific DNA sequence in the genome [3, 4]. DNA methylation is catalyzed by Dnmts mainly Dnmt1, Dnmt3a and Dnmt3b [5]. The Dnmts share a C-terminal methyltransferase catalytic domain, which transfers a methyl group from S-adenosyl methionine to the 5-carbon position of CpG cytosines in double-stranded DNA [5–7]. Methylation of the promoter region of a gene blocks the binding of transcription factors, thus inhibits the gene transcription [6].

Stroke is the second leading cause of death worldwide, and compelling evidence supports a role for DNA methylation in this process [3]. DNA methylation might exacerbate neuronal injury due to upregulation of Dnmts. A DNA methylation inhibitor 5-aza-2’-deoxycytidine (decitabine) conferred protection in stroke [4]. The demethylating agent, decitabine ameliorated ischemia-induced neuronal death by inhibiting DNA methylation activity [3, 8].

Astrocytes are the most abundant glial cell types in the central nervous system (CNS). They are involved in focal ischemic stroke. They contribute to the pathological features of focal ischemic stroke such as reactive astrogliosis and glial scar formation [9, 10]. Glial scar has long been considered a barrier to axonal regeneration after neural lesion [11]. But recent studies have shown that glial scar formation aids rather than stops CNS axon regeneration [12, 13].

DNA methylation is a critical cell-intrinsic determinant of astrocyte differentiation in the brain [14]. DNA methylation in astrocytes appears to be altered in numerous neurological disorders, which has broad implications for controlling both the neuroprotective and neurodegenerative functions of astrocytes [15].

In this study, we hypothesized that inhibiting DNA methylation by decitabine might reduce the proliferation of astrocytes by promoting some gene expression in astrocytes of rats with ischemic stroke. Experiments were performed to test whether intervention with decitabine could ameliorate stroke and reduce astrogliosis. The mechanism of decitabine was investigated.

## Materials and methods

### Animals and ethics statement

Male Sprague-Dawley rats weighing about 300–320 g were purchased from the experimental animal center of China Medical University (Shenyang, China). They were fed ad libitum under conditions of temperatures ranging between 22–24°C in a house with controlled 12-hour light and 12-hour dark cycle. All procedures were approved by the animal Ethics Committee of China Medical University (Approval Number: 201903278). All experimental procedures were conducted according to the guidelines and regulations for the care and use of laboratory animals by the Ethics Committee of China Medical University. This study was carried out in compliance with the ARRIVE guidelines.

### MCAO model and experimental groups

Rats were anesthetized with isoflurane mixed with oxygen and then subjected to middle cerebral artery occlusion (MCAO) as described previously [16, 17]. Briefly, the right common carotid artery (CCA) and external carotid artery were exposed and detached from the surrounding tissue carefully. A monofilament with a diameter of 0.47 ± 0.02 mm (RWD, China) covered with smooth silica gel was inserted (about 20 mm) via the CCA into the right internal carotid artery and into the circle of Willis, occluding the right middle cerebral artery (MCA). After 60 min of MCAO, the monofilament was carefully pulled out to initiate reperfusion. When the rats woke up, left-side rotation of the rats confirmed the successful establishment of a MCAO model. A sham operation was performed in the similar manner, with the omission of MCAO. Rats were randomly divided into 4 groups (n = 36/group): (1) sham + vehicle (S + V) group, (2) sham + decitabine (S + D) group, (3) MCAO + vehicle (M + V) group, and (4) MCAO + decitabine (M + D) group. Decitabine was bought from Selleck Chemicals (USA) and was dissolved in normal saline and intraperitoneally injected (0.12 mg/kg) immediately after MCAO in S + D and M + D groups. Equal amount of normal saline was intraperitoneally injected into rats of S + V and M + V groups.

### Assessment of infarct volume

The brain infarct volume was evaluated 4 days after MCAO. Rats (n = 6/group) were anesthetized and the brains were quickly excised and fast frozen at -20°C for 30 min. The brains were cut into 2-mm-thick coronal sections from the frontal pole to the occipital pole to yield 5 slices with a brain mold (RWD, China). The slices were stained with 2% 2, 3, 5-triphenyltetrazolium chloride (Sigma-Aldrich) at 37°C for 20 min in darkness and then fixed in 4% paraformaldehyde for more than 2 h. Normal tissue stained red while the infarct tissue appeared white. The infarct area was selected and analyzed by Image J software (NIH). The possible interference of brain edema in the infarct volume was corrected using standard methods (contralateral hemisphere volume–volume of non-ischemic ipsilateral hemisphere), with infarct volume expressed as a percentage of that of the contralateral hemisphere [17, 18].

### Immunofluorescent and EdU staining

Immunofluorescent staining was performed as previously described [17]. BeyoClick EdU cell proliferation kit with alexa fluor 488 was purchased with Beyotime in Shanhai. EdU was intraperitoneally injected (50 mg/kg) 2 hours before taking brain tissue. On day 4 after MCAO, the rats (n = 6/group) were anesthetized and perfused with 0.9% normal saline and 4% paraformaldehyde. Brains were excised and placed in 4% paraformaldehyde solution for more than 24 h and dehydrated in 30% sucrose solution. Coronal brain slices around infarction area were sectioned at 10 μm thickness with a freezing microtome (Leica CM1950, Germany) and rinsed using 0.01 M PBS 3 times for 5 min each. They were then blocked with 5% goat serum or 5% BSA in 0.3% triton X-100 for 60 min at room temperature. The sections were first incubated with primary antibodies including mouse anti-NeuN Antibody (1:1000; mab377, Sigma-Aldrich), rabbit anti-5-methylcytosine (5-mC) antibody (1:200; ab214727, Abcam), rabbit anti-ki67 antibody (1:250; ab16667, Abcam), rabbit anti-p27 antibody (1:200; ab32034, Abcam), goat anti-GFAP antibody (1:500; ab53554, Abcam) at 4°C overnight in the refrigerator. The sections were then incubated with secondary antibodies including goat anti-mouse IgG (1:2000; 8890S, CST), goat anti-rabbit IgG (1:2000; 4412S, CST), and donkey anti-goat IgG (1:2000; ab6949, Abcam) for 1 h at room temperature. The nuclei were stained with DAPI (C1005, Beyotime, Shanghai, China). Fluorescence images were captured by Nikon Eclipse 80i (Japan).

The co-localization of astrocyte marker GFAP with 5-mC, p27, ki67 or EdU was evaluated by using Image J. At least 100 cells were examined to calculate the percentage of cells with cytoplasmic localization. Five animals in each group were used for the analysis, with five images from each animal [19]. For NeuN staining, five typical slices were chosen to represent an animal and five visual fields at 20 X magnification in each slice were captured for the counting.

### Western blot analysis

Western blot was performed as previously described [17]. On day 4 after MCAO, the rats (n = 6/group) were anesthetized and the ipsilateral peri-infarct cortex was isolated on ice. Proteins were extracted from ipsilateral cortical tissue using RIPA lysis buffer, phenylmethanesulfonyl fluoride (PMSF), and phosphatase inhibitor. Equal amounts of protein were separated via 10% SDS-PAGE and then transferred to PVDF membranes, and blocked by 5% skim milk. The following primary antibodies were used: rabbit anti-β-actin (1:5000; 20536-1-AP, Proteintech, Wuhan, China), rabbit anti-GFAP (1:5000; 16825-1-AP, proteintech, Wuhan, China), and mouse anti-NeuN Antibody (1:5000; mab377, Sigma-Aldrich). All antibodies were diluted with 5% skim milk in TBST, and incubated at 4°C overnight. On the following day, the membrane was incubated with secondary goat anti-rabbit IgG and goat anti-mouse IgG (1:1000; Beyotime, Shanghai, China) labeled by horseradish peroxidase was added and incubated for 1 h at room temperature. Chemiluminescence imaging system (ChemiDoc TM XRS+, Bio-Rad, USA) was used for ECL luminescence. The Image J software was used to analyze the protein bands, and β-actin served as the internal control.

### DNA methylation analysis

DNA methylation analysis was performed by dot blot [20]. On day 4 after MCAO, genome DNA was extracted from the ipsilateral peri-infarct cortex with the kit (D0061, Beyotime, Shanghai, China) and denatured at 99°C for 5 min, and immediately put on the ice. Dot blot procedure was performed following the protocols provided by Abcam. The single-stranded DNA was measured, and 2 μl of the sample (100 and 50 ng/μl) were dropped onto a nitrocellulose membrane at the center of the grid while ensuring the solution penetrated a small area (usually 2–4 mm diameter) by applying it slowly. The membrane was dried and blocked by 5% skim milk in TBST for 1 h at room temperature. The membrane was incubated with primary rabbit anti-5-methylcytosine (5-mC) antibody (1:2000; ab214727, Abcam) at 4°C overnight. Subsequently, the membrane was incubated with secondary goat anti-rabbit IgG (1:1000; Beyotime, Shanghai, China) labeled with horseradish peroxidase for 1 h at room temperature. The ECL luminescence was measured using a Chemiluminescence imaging system (ChemiDoc TM XRS+, Bio-Rad).

### Astrocyte culture and treatment

Astrocytes were cultured as previously described [21]. Briefly, cerebral cortical astrocytes were obtained from neonatal SD rats. The neonatal rats were rapidly euthanized, and the whole brains were transferred into ice-cold D-Hanks. The pia mater was stripped under a microscope and the brain cortices were separated carefully. Then the cortices were digested with 0.25% trypsin at 37°C for 15 min. Cells were mechanically dissociated in a culture medium containing 10% fetal bovine serum (FBS) and plated in culture dishes at a density of 5 × 10^5^ cells/ml. The cells were cultured in a humidified cell incubator at 37°C of 5% CO_2_. Astrocytes were treated with different concentrations of decitabine (0 μM, 5 μM, and 10 μM) for 24 h and then used for qRT-PCR.

### Quantitative reverse transcription real-time polymerase chain reaction (qRT-PCR)

QRT-PCR was performed as previously described [22, 23]. Briefly, total RNAs were isolated and purified with TRIzol reagent (Invitrogen, USA). Complementary DNA was synthesized from the total RNA using HiScript II Q RT Super Mix kit (Vazyme, Nanjing, China). QPCR was carried out with Step One Plus Real-Time PCR System (Applied Biosystems) using ChamQ Universal SYBR qPCR Master Mix kit (Vazyme, Nanjing, China). PCR conditions were 95°C for 30 s, followed by 40 cycles at 95°C for 10 s and 60°C for 30 s. PCR primers used were: p27, 5’-TCTCAGGCAAACTCTGAGGA-3’ (F) and 5’-CTTCCTCATCCCTGGACACT-3’ (R) [24]; β-actin, 5’-ACATCCGTAAAGACCTCTATGCCAACA-3’ (F) and 5’-GTGCTAGGAGCCAGGGCAGTAATCT-3’ (R). The mRNA expression level was normalized to the expression level of the housekeeping gene β-actin. All analyses were performed by using the 2^−ΔΔCt^ method [25].

### Statistical analysis

All the data are presented as mean ± SD, and were analyzed by one-way ANOVA, followed by Tukey’s test using GraphPad Prism 5 software. A p value of < 0.05 was considered statistically significant. Mean values for infarct volume between 2 groups were compared with unpaired t-test.

## Results

### Decitabine reduced DNA methylation and infarct volume after MCAO

**Fig 1:**
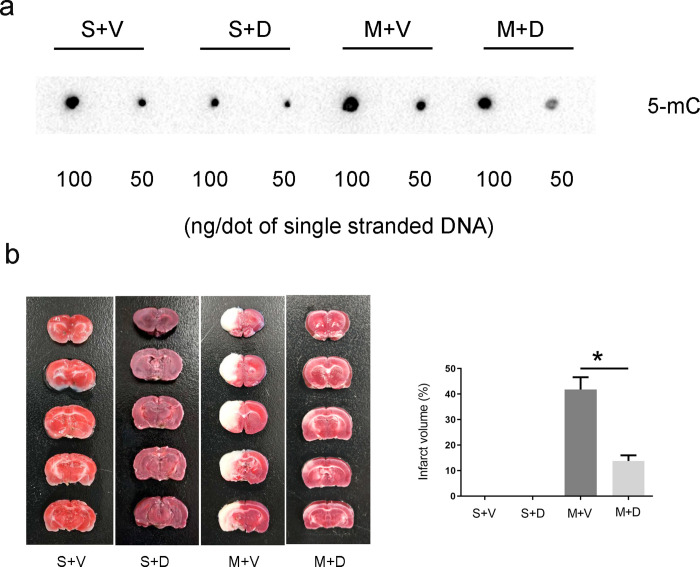
Decitabine treatment reduces DNA methylation and brain infarct volume on day 4 after MCAO. (a) Dot blot with anti-5-mC shows the DNA methylation level in each group on day 4 after MCAO. Two sample DNA concentrations (100 and 50 ng/μl) are used. (n = 6/group). (b) TTC staining shows the brain infarct volume in each group with normal tissue stained red and the infarct tissue remaining white (n = 6/group, *p < 0.05, unpaired t-test).

Previous studies have shown that Dnmts and DNA methylation are upregulated in the brain of stroke models including MCAO, and 5-aza-dC or decitabine can reduce the infarct volume and neuronal death in these models [1, 4, 26]. To clarify this finding, we first investigated whether decitabine lowered genome DNA methylation on day 4 after MCAO. Changes in DNA methylation were tested by dot blot using anti-5-mC antibody. The expression of 5-mC in the M + D group was significantly lower than that in the M + V group at the same concentration of decitabine (Fig 1A). Similar results were obtained between S + D and S + V group (Fig 1A). The infarct volume in the M + D group was markedly lower than in the M + V group (41.75 ± 4.21 versus 12.75 ± 1.92, unpaired t-test p < 0.05, Fig 1B).

### Decitabine reduced DNA methylation in astrocytes

**Fig 2:**
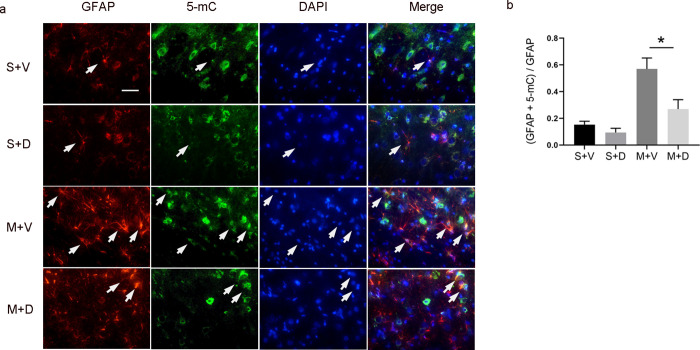
Decitabine treatment reduces DNA methylation in astrocytes. (a) The immunofluorescence staining demonstrates the co-localization of astrocyte marker GFAP (red) with 5-Mc (green) and nuclear marker DAPI (blue) in the peri-infarct area on day 4 after MCAO between M + V and M + D groups. Scale bar = 20 μm. (b) Quantitative analysis shows the co-localization difference of 5-mC with GFAP between M + V and M + D groups, n = 6/group, *p < 0.05 by one-way ANOVA plus Tukey’s test.

DNA methylation was present in all cells. To determine the change of DNA methylation in astrocytes, we double-stained the tissue sections with antibodies against DNA methylation marker 5-mC and astrocyte marker GFAP (Fig 2A). The quantitative analysis showed that the level of DNA methylation in astrocytes in M + D group was markedly lower than that in M + V group (0.55 ± 0.09 versus 0.23 ± 0.08, p < 0.05, Fig 2B).

### Decitabine upregulated mRNA and protein expression of P27

**Fig 3:**
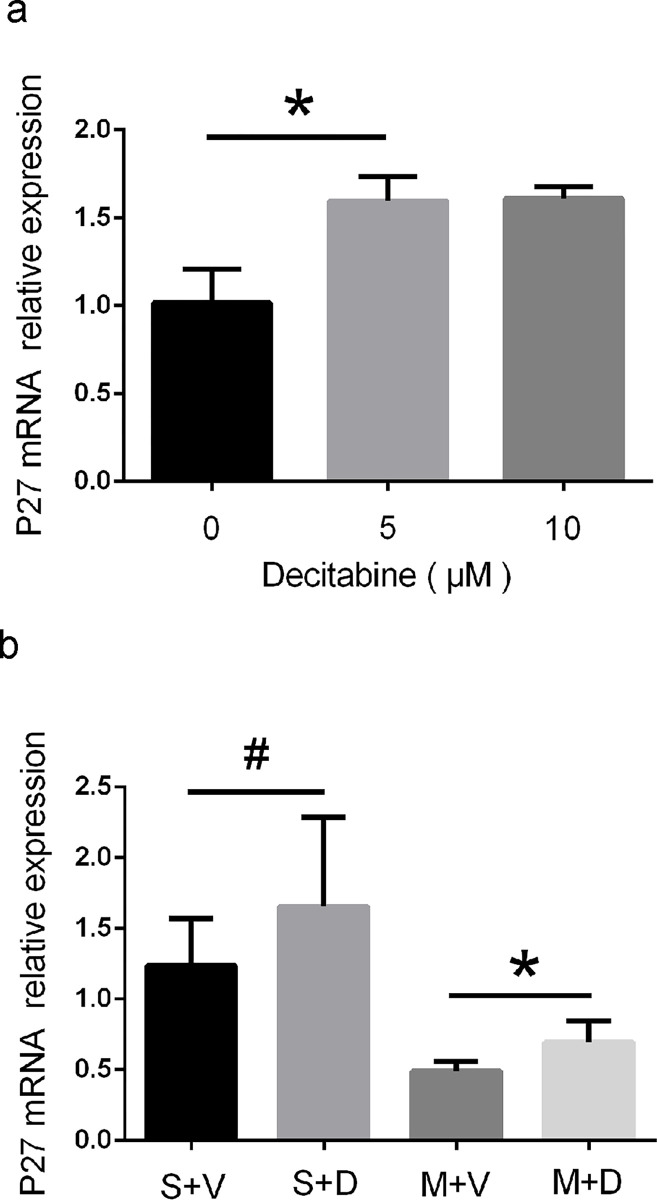
Quantitative RT-PCR shows decitabine upregulates the mRNA level of p27 in vitro and in vivo. (a) Cultured astrocytes are treated with 0 μM, 5 μM, and 10 μM decitabine (n = 6/group, *p < 0.05 by one-way ANOVA plus Tukey’s test). (b) Decitabine is intraperitoneally injected to the rats after MCAO, qRT-PCR is performed on day 4 (n = 6/group, ^#^p < 0.05 and *p < 0.05 by one-way ANOVA plus Tukey’s test).

**Fig 4:**
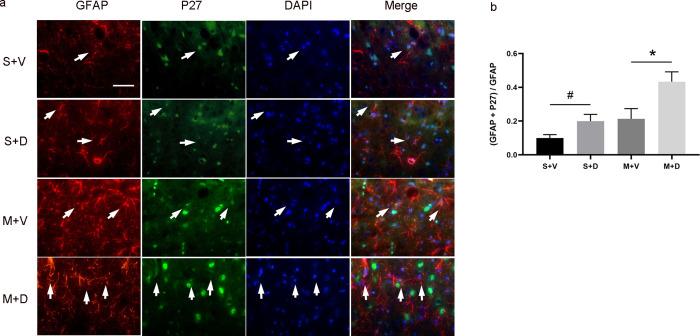
Decitabine treatment increases the expression of p27 in astrocytes. (a) The immunofluorescence staining demonstrates the co-localization of astrocyte marker GFAP (red) with p27 (green) and nuclear marker DAPI (blue) in the peri-infarct area on day 4 after MCAO. Scale bar = 20 μm. (b) Quantitative analysis shows the p27 expression changes with GFAP in each group, n = 6/group, *p < 0.05 by one-way ANOVA plus Tukey’s test.

Cdkn1b (p27) gene encodes a cyclin-dependent kinase inhibitor which controls the cell cycle causing arrest at G1 phase. To test the effect of decitabine on p27 gene, we first added decitabine into the astrocytic culture solution at different concentrations. The results showed that p27 mRNA in the group subjected to 5 μM or 10 μM of decitabine was significantly higher than the group treated with 0 μM of decitabine (1.02 ± 0.19 versus 1.60 ± 0.14, p < 0.05, Fig 3A). In vivo, the p27 mRNA expression in the S + D and M + D groups was markedly higher than in the S + V and M + V groups (1.66 ± 0.63 versus 1.24 ± 0.34, p < 0.05; 0.69 ± 0.15 versus 0.49 ± 0.07, p < 0.05, Fig 3B). To test the co-localization of GFAP with p27 protein, double immunofluorescent staining of GFAP (red) and p27 (green) was performed (Fig 4A). The quantitative analysis of co-localization of GFAP with p27 protein for astrocytes showed that the expression level of p27 in astrocytes was significantly higher in S + D and M + D groups than those in S + V and S + D groups (0.20 ± 0.04 versus 0.10 ± 0.02, p < 0.05, 0.43 ± 0.06 versus 0.21 ± 0.06, p < 0.05, Fig 4B).

### Decitabine reduced astrocyte proliferation and increased quantity of neurons

**Fig 5:**
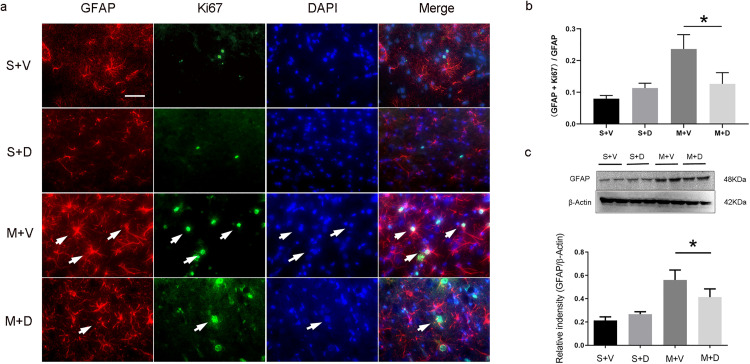
Decitabine treatment reduces the expression of ki67 in astrocytes. (a) The immunofluorescence staining demonstrates the co-localization of astrocyte marker GFAP (red) with ki67 (green) and nuclear marker DAPI (blue) in the peri-infarct area on day 4 after MCAO. Scale bar = 20 μm. (b) Quantitative analysis shows the co-localization difference of ki67 with GFAP in 4 groups, n = 6/group, *p < 0.05 by one-way ANOVA plus Tukey’s test. (c) Quantitative analysis of western blot shows protein expression of GFAP in each group on day 4 after MCAO, n = 6/group, *p < 0.05 by one-way ANOVA plus Tukey’s test.

**Fig 6:**
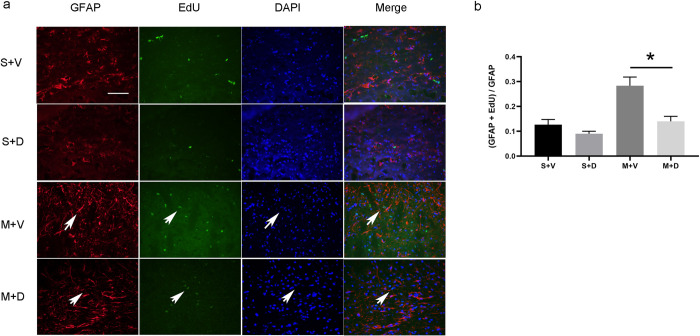
Decitabine treatment reduces the proliferation of astrocytes. (a) It demonstrates the co-localization of astrocyte marker GFAP (red) with EdU (green) and nuclear marker DAPI (blue) in the peri-infarct area on day 4 after MCAO. Scale bar = 20 μm. (b) Quantitative analysis shows the co-localization difference of EdU with GFAP in 4 groups, n = 6/group, *p < 0.05 by one-way ANOVA plus Tukey’s test.

**Fig 7:**
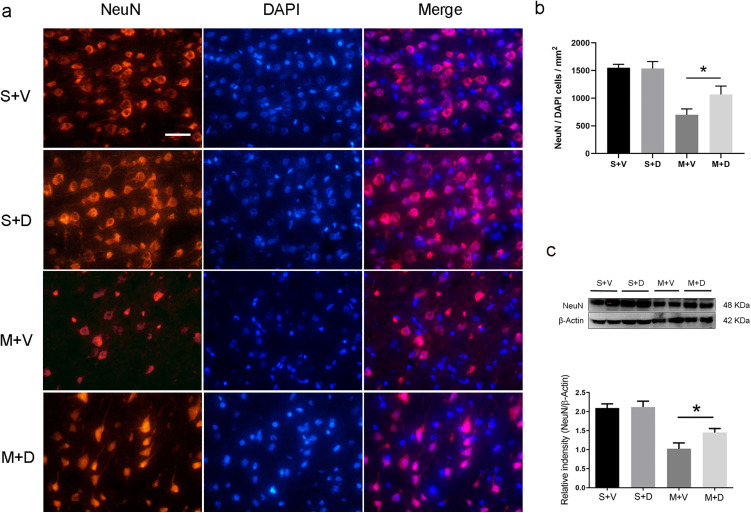
Decitabine treatment increases total quantity of neurons on day 4 after MCAO. (a) The immunofluorescence staining demonstrates the co-localization of neuronal marker NeuN (red) and nuclear marker DAPI (blue) in the peri-infarct area on day 4 after MCAO. Scale bar = 20 μm. (b) Bar graph shows the surviving neurons in the peri-infarct area in each group, n = 6/group, *p < 0.05 by one-way ANOVA plus Tukey’s test. (c) Quantitative analysis of western blot shows the protein expression of NeuN in each group on day 4 after MCAO, n = 6/group, *p < 0.05 by one-way ANOVA plus Tukey’s test.

To test if the protective effect of decitabine in the stroke model was caused by changed in astrocytes, we examined the proliferation of astrocytes. Ki67 and EdU are biomarker of cell proliferation [27, 28]. To test the co-localization of GFAP with ki67 protein and EdU, double immunofluorescent staining of GFAP (red) with ki67 (green) was performed (Fig 5A). The quantitative analysis of co-localization of GFAP with ki67 for astrocytes showed that the expression level of ki67 in astrocytes was lower in M + D group than that in M + V group (0.13 ± 0.04 versus 0.24 ± 0.05, p < 0.05, Fig 5B). Western blot analysis showed the expression level of GFAP was significantly lower in the M + D group than that in the M + V group (0.59 ± 0.06 versus 0.42 ± 0.08, p < 0.05, Fig 5C). Double immunofluorescent staining of GFAP (red) with EdU (green) was performed (Fig 6A). The quantitative analysis of co-localization of GFAP with EdU for astrocytes showed that the expression level of EdU in astrocytes was lower in M + D group than that in M + V group (0.14 ± 0.03 versus 0.28 ± 0.04, p < 0.05, Fig 6B). Analysis of immunofluorescent staining of neuronal marker NeuN and DAPI (Fig 7A) indicated that there were more neurons in the M + D group than that in the M + V group (1100 ± 87 versus 710 ± 50, p < 0.05, Fig 7B). Western blot analysis showed the expression level of NeuN was significantly higher in the M + D group than that in the M + V group (1.46 ± 0.05 versus 1.05 ± 0.06, p < 0.05, Fig 7C).

## Discussion

In this study, we demonstrated that DNA methylation inhibitor, decitabine attenuated ischemia/reperfusion after MCAO by repressing DNA methylation and reducing proliferation of astrocytes. We speculated that DNA methylation might occur in the regulatory regions of p27 gene and control its expression in astrocytes.

As one of the epigenetic mechanisms, DNA methylation plays a role in the pathophysiology of stroke [3]. In a previous study, DNA methylation was significantly higher in ischemic cortex than in the contralateral tissues 12 h after MCAO [1]. The present study found that DNA methylation levels in M + V group were also more than in S + V group (Fig 1A). Our data were consistent with previous studies.

We observed that the increase of DNA methylation occurred in astrocytes after MCAO (Fig 2). However, we didn’t examine the change of DNA methylation in neurons and microglia. We speculated that the change of DNA methylation in microglia was similar to that in astrocytes after MCAO. A previous study demonstrated the increase of DNA methylation mainly occurred in hypoxia-induced astrocytes but not in neurons [29]. The effect of DNA methylation in neurons after MCAO will be our next study.

P27 has been implicated in the control of cell cycle progression and cell proliferation by binding and regulating nuclear cyclin-dependent kinases [30, 31]. It has also been associated with astrocytes and microglia proliferation [32]. Adenovirus vectors-mediated p27 overexpression inhibited astrocyte proliferation following cortical injury which lasted longer in p27-deficient mice than in wild-type mice [33]. In this study, we observed that hypomethylation induced by decitabine increased p27 expression to decrease the astrocytes proliferation. The effect of DNA methylation in astrocyte differentiation were investigated by many studies. DNA methylation in astrocytes has broad effects for controlling both the neuroprotective and neurodegenerative qualities of astrocytes [15].

Astrocyte activation and scar formation induced by stroke can be barriers to axonal regeneration after stroke [34]. However, functions of reactive astrocytes are still a subject of debate, with some evidence supporting their obstructive effects, and others supporting beneficial effects in CNS recovery [35]. This discrepancy may be attributed to astrocytes in different phases of the injury. In acute phase, reactive astrocytes may limit the damage by sealing the lesion site, restore homeostasis, preserve spared tissue, and regulate immune responses. In chronic phase, reactive astrocytes eventually form glial scar in the infarct border zone that demarcates the ischemic core (infarction) from healthy tissue, blocking the regrowth of nerves [36]. Therefore, it is necessary to clarify the mechanism and function of astrocytes in different phases of injury to get an idea strategy for CNS recovery.

However, in our study, we found that mild activation of astrocytes resulted in better results than excessive activation of astrocytes after MCAO. We have known that it would produce worse outcomes without the protection of glial scar in MCAO. So, the dose of decitabine we used in the study was low and it could only inhibit glial scar slightly. Recent gene profiling studies proposed that there are at least two types of reactive astrocytes, depending on the stimuli inducing CNS injury, called A1 and A2, which may be harmful or beneficial in neuroinflammation and ischemia, respectively [13]. P27 gene might repress the proliferation of astrocytes and accelerate recovery after ischemic brain injury.

The present study found that the total number of neuronal survival in M + D group was more than in M + V group (Fig 7). We thought it was potential therapeutic benefit of suppressing DNA methylation.

In conclusion, p27 gene expression was increased by inhibiting DNA methylation in astrocytes and reduced proliferation of astrocytes. Decitabine reduced infarct volume after ischemic stroke.

## Supporting information

S1 File Raw Images. (PDF)
