# Correction: Decitabine attenuates ischemic stroke by reducing astrocytes proliferation in rats

**DOI:** 10.1371/journal.pone.0355796

**Published:** 2026-08-10

**Authors:** The PLOS One Staff

## Notice of Republication

This article was republished on July 24^th^, 2026, to include the correct images for Figs 5 and 7 and missing supplemental images. The publisher apologizes for the errors. Please download this article again to view the correct version. The originally published, uncorrected article and the republished, corrected articles are provided here for reference.

## Supporting information

S1 File Originally published, uncorrected article. (PDF)

S2 File Republished, corrected article. (PDF)
