# Supplementary figures and images for: Decitabine attenuates ischemic stroke by reducing astrocytes proliferation in rats
Source: PLoS One. 2022 Aug 2;17(8):e0272482. doi: 10.1371/journal.pone.0272482 (PMC9345475; doi:10.1371/journal.pone.0272482)

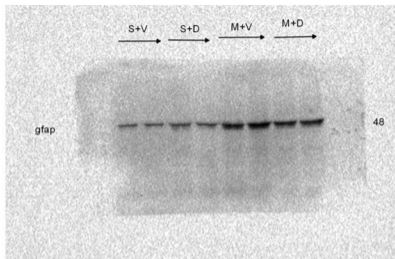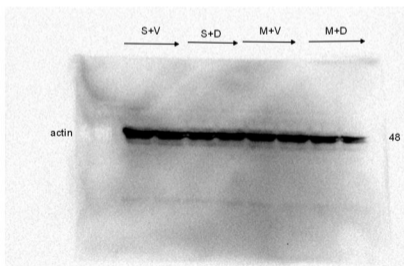

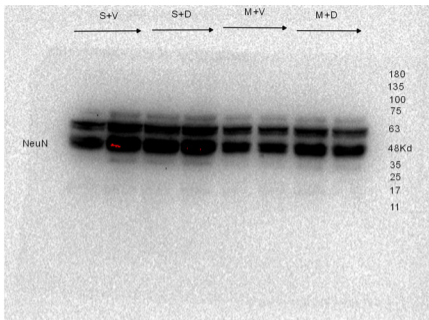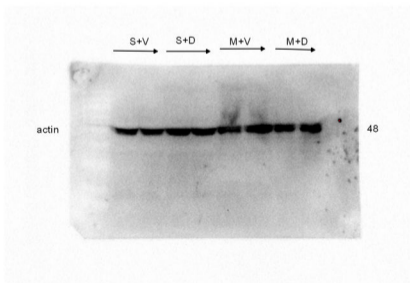

Supplement: S1 File — (PDF) [file pone.0272482.s001.pdf]
