# Supplementary material for: Decitabine attenuates ischemic stroke by reducing astrocytes proliferation in rats
Source: PLoS One. 2022 Aug 2;17(8):e0272482. doi: 10.1371/journal.pone.0272482 (PMC9345475; doi:10.1371/journal.pone.0272482)
Supplement: Attachment — Submitted filename: Respose to reviewers.docx [file pone.0272482.s002.docx]

Dear Editors and Reviewers:

We thank you very much for giving us an opportunity to revise our manuscript, we appreciate editor and reviewers very much for their positive and constructive comments and suggestions on our manuscript entitled **“**Decitabine Attenuates Ischemic Stroke by Reducing Astrocytes Proliferation in Rats”**.** We have studied reviewer’s comments carefully and have tried our best to revise our manuscript according to the comments. We would like to express our great appreciation to you and reviewers for comments on our manuscript.

Reviewer #1: Overall, this was an excellent manuscript. The methods were clear, concise and complete. The experimental plan was well thought out and addressed an important problem. The introduction provided sufficient background to put the research into context and introduced the drugs and gene products that would be used or measured in the study. The figures were clearly understandable, although some comparisons were missing (see below). There are a few relatively minor problems which I would like to see addressed, primarily, with respect to issues raised by the data which were not addressed in the discussion. I would like to see the discussion optimized to encourage others to follow up on this work.
Issues to address:

1) The second to the last line of the introduction stated: “Experiments were performed to test whether intervention with decitabine could ameliorate stroke and improve astrogliosis.” This statement is ambiguous. Given that astrogliosis is believed to have both positive and negative effects on ischemic stroke, it is not clear whether “improving” astrogliosis would involve an increase, decrease, or change in timing of astrogliosis. I would suggest replacing “improve” either with a more neutral word, such as “influence”, or a more specific description of your expectation, such as “reduce”.

**Response:** Thanks for your valuable comment. We have used “reduce” instead of “improve”.

2) Similarly, the second to the last sentence of the discussion states: “In conclusion, dicitabine improved p27 gene expression ...” Once again, it is not clear that increasing P27 expression represent “an improvement”. It would be better to state simply that P27 expression was increased. Note also that decitabine was misspelled here.

**Response:** Thanks for your valuable comment. I have revised the manuscript with “p27 expression was increased”, and corrected spelling mistake of decitabine.

3) Figure 1B shows very different gross coloring of stained brain sections in the S+D condition. I would have liked to see a higher magnification of this tissue or some explanation for the gross differences between the appearance of sham surgery brain of the vehicle and decitabine treated animals. Was there diffuse brain hemorrhaging or macrophage invasion in the decitabine treated animals? This might imply a negative effect of the drug on the blood-brain-barrier which would complicate interpretation of the effects of decitabine on astrocytes

**Response:** Thanks for your valuable comment. Figure 1B shows the TTC staining. TTC was purchased from Sigma-Aldrich and was used repeatedly. As the number of staining increased, the gross coloring was changed from light red to deep red. It didn’t affect the calculation of infarct area. There were no differences between the appearance of sham surgery brain of the vehicle and decitabine treated animals. We observed that there was no diffuse brain hemorrhaging or macrophage invasion in the decitabine treated animals under safe dose.

4) The green (FITCI) staining for P27 in figure 3 appears to have rather high diffuse background staining. This figure would be easier for people less familiar with immunocytochemistry to understand if some of the background was thresholded out and contrast enhanced for the GFAP and DAPI staining.

**Response:** Thanks for your valuable comment. We have revised the high diffuse background staining of P27 and DAPI.

5) For the benefit of electrophysiologists and molecular biologist, it would be useful to note in the figure legends that, for example P27 staining was also observed in cells that were not astrocytes. Otherwise, some people may be confused about why some hot spots are pointed out with arrow, while much larger hot spots are not.

**Response:** Thanks for your valuable comment. P27 has been implicated in the control of cell cycle progression and cell proliferation. P27 is expressed in not only astrocytes, but also in microglia, oligodendrocytes, endothelial cells and so on. In this study, we only focus on the changes and effects of P27 in astrocytes. In future, we will check the changes of P27 in other cells of the brain.

6) The authors understandably have focused on differences between decitabine treated and vehicle treated conditions within the sham and stroke model conditions, but comparisons between sham and ischemia condition may also be informative.

**Response:** Thanks for your valuable comment. Contrast to sham, in ischemic condition we found that the methylation increased in astrocytes, p27 mRNA expression decreased in astrocytes, and astrocytes activate and proliferate. Because of focusing on the effects of decitabine on ischemia, we think less about comparisons between sham and ischemia condition.

7) The decrease in overall DNA methylation in the MCAO vehicle treated condition, relative to the sham condition while staining of tissue showed an increase in DNA methylation in astrocytes should be briefly addressed. The apparent discrepancy between global methylation levels and methylation levels in astrocytes suggests that at this timepoint, methylation levels are significantly decreased in some, or all other cell types in the brain. A decrease in methylation levels in endothelial cells, microglia, or neurons, might be as significant as a sustained increase in methylation levels in astrocytes.

**Response:** Thanks for your valuable comment. We repeated the experiment after increasing the sample size and found the level of 5-mc increased after MCAO assuredly. It meaned that both the global methylation levels and methylation levels in astrocytes increased. We have revised it in the Figure.1.

8) Figure 6 shows that the numbers of NueN positive cells were increased in decitabine treated MCAO animals compared to MCAO animals with vehicle treatment. This is the best evidence the authors have for potential therapeutic benefit of suppressing DNA methylation, since it implies increased neuronal survival. However, this data is not even mentioned in the Discussion. It should be

**Response:** Thanks for your valuable comment. We have mentioned it in the discussion in the penultimate paragraph. “The present study found that the total number of neuronal survival in M + D group was more than in M + V group (Fig 6). We thought it was potential therapeutic benefit of suppressing DNA methylation.”

9) At the beginning of the introduction the authors state that: “dectabine attenuated ischemia/reperfusion after MACO by repressing DNA methylation and reducing the proliferation of astrocytes”. While this is the simplest explanation for the observed reduction in infarct volume, direct causation has not been established. One of the unavoidable complication of systemic delivery of a drug and waiting several days for results, is the possibility that the drug (or its metabolic breakdown products) could be having an indirect effect on the parameter you are measuring through other cells. Please restate this.

**Response:** Thanks for your valuable comment. Yes, it was one of the unavoidable complication of systemic delivery of a drug. At present, we only drew the conclusion that decitabine attenuated ischemia/reperfusion after MCAO by repressing DNA methylation. The effects of decitabie on other cells had not been measured. This was our shortcoming.

10) An additional issue that should be briefly noted is that the short-term nature of these experiments does not allow for evaluation of the long-term effects of suppressing astrogliosis on the functional outcome. It may be that without the containment effects of the glial scar, diffuse brain damage might have extended to both sides of the brain, resulting in worse outcomes.

In short, this very good paper, but I would like to see the Discussion clarified a bit and expanded cover matters hinted at by the data, but not addressed in the current draft of the Discussion

**Response:** Thanks for your valuable comment. We have known that it would produce worse outcomes without the protection of glial scar in MCAO. So, the dose of decitabine we used in the study was low and it could only inhibite glial scar slightly. There was still some glial scar to prevent diffuse brain damage. This is supplemented in the discussion.

Reviewer #2: The present study “Decitabine attenuates ischemic stroke by reducing astrocytes proliferation in rats” by Xu Zhang and colleagues investigated whether DNMT inhibitor, decitabine may reduce the proliferation of astrocytes and ameliorate stroke by promoting p27 gene expression. Though the subject is intriguing and the authors presented novel data, there are several concerns in the design of methods and interpretation of the results.

1. Single dose of 0.12mg/kg of decitabine was used in this study, which seems to be rather low dosage in comparison with other studies. How did you decide the dosage of decitabine?

**Response:** Thanks for your valuable comment. We have found that the dose of 0.4 - 0.5 mg/kg facilitated death of MCAO rats. Glial scar can limit the diffuse brain damage. Too large dose decitabine can reduce gliar scar and facilitate death. The dose of 0.10 -0.15 mg/kg can ruduce infarct volume. We found that inhibiting glial scar slightly could bring better results than excessive inhibiting glial scar.

2. Regulation of gene expression by DNA methylation reported to occur in the regulatory regions with rich CPG islands. Data on the CPG island proportion of regulatory regions of p27 genes need to be discussed, if available.

**Response:** Thanks for your valuable comment. We are sorry that we didn’t finish the study about data on the CPG island proportion of regulatory regions of p27 genes.

3. Compared to sham control, the level of 5 mc after MCAO is low (Fig 1a), which conflicts with the previous reports that DNA methylation is increased after stroke. Authors need to explain this contradictory finding.

**Response:** Thanks for your valuable comment. We repeated the experiment after increasing the sample size and found the level of 5-mc increased after MCAO assuredly. We have revised it in the Figure.1.

4. 5-mc immunopositive staining shows that the proportion of astrocytes among the total immunopositive cells is not high. It is necessary to determine other cells that the 5-mC signal does not colocalize with GFAP.

**Response:** Thanks for your valuable comment. We have found that neuron colocalized with 5-mC signal by immuofluorescent staining. A previous study (below) demonstrated the increase of DNA methylation mainly occurred in hypoxia-induced astrocytes but not in neurons. So, we focused on the changes of methylation of astrocytes in our study.

Yang Q, Wu X, Sun J, Cui J, Li L. Epigenetic Features Induced by Ischemia-Hypoxia in Cultured Rat Astrocytes. Mol Neurobiol. 2014;53(1):436-45. doi: 10.1007/s12035-014-9027-8.

5. In the acute phase of ischemic stroke, the proliferation of astrocytes in the peri-infarct area is reported to be helpful to sealing the site of injury and controlling the local immune response. On the contrary, authors suggested that reducing the proliferation of astrocytes 4 days period after MCAO with decitabine has a neuroprotective effect. Additional verification and consideration of the meaning of acute phase astrocyte proliferation are needed.

**Response:** Thanks for your valuable comment. We have known that in the acute phase astrocyte proliferation were needed because glial scar was helpful to sealing the site of injury and controlling the local immune reaponse. So, the dose of decitabine we used in the study was low and it could only inhibite glial scar slightly. There was still glial scar to prevent diffuse brain damage.

6. Since the level of GFAP increases not only in proliferation but also in the activation of astrocytes, additional experimental validation such as BrdU incorporation is needed in addition to simple expression of p27 and ki67 to prove changes of astrocyte proliferation.

**Response:** Thanks for your valuable comment. EdU is a modified way contrast to BrDU. and we used EdU in our study. We showed it in Figure. 6.
